# Visceral Leishmaniasis and HIV Coinfection: Time for Concerted Action

**DOI:** 10.1371/journal.pntd.0003023

**Published:** 2014-08-28

**Authors:** Johan van Griensven, Ed E. Zijlstra, Asrat Hailu

**Affiliations:** 1 Department of Clinical Sciences, Institute of Tropical Medicine, Antwerp, Belgium; 2 Rotterdam Centre for Tropical Medicine, Rotterdam, the Netherlands; 3 School of Medicine, Addis Ababa University, Addis Ababa, Ethiopia

Visceral leishmaniasis (VL) is a vector-borne protozoan disease caused by species of the *Leishmania donovani* complex. It is a global health problem, with an estimated annual incidence of 200,000–400,000 cases [1]. VL caused by *L. infantum* (*chagasi*) is mainly prevalent in Latin America and the Mediterranean region, whereas *L. donovani* causes VL in the Indian subcontinent and eastern Africa [2].

HIV coinfection of VL has been identified as one of the emerging challenges for VL control [3]. HIV infection of *Leishmania*-exposed individuals dramatically increases the risk of progression from asymptomatic infection towards disease (VL) and, conversely, VL accelerates HIV disease progression. Whereas HIV fuelled the re-emergence of VL in Southern Europe in the 1990s, this problem is now severe in some areas of eastern Africa, particularly in northern Ethiopia. In earlier studies, up to 40% of VL patients were HIV coinfected in this region [3]–[5]. A more recent study found a prevalence of 18% [6]. Seasonal migrant workers traveling to large agricultural fields in northwestern Ethiopia are most at risk. Due to the widespread commercial sex practice in the region, the risk of dual infection of the migrant workers is high. The problem is also on the rise in South America, with coinfection rates reaching 6% in 2011 [7]. This has been linked to the increased geographical overlap between the two infections, whereby VL is increasingly reported in peri-urban settings [8]. Importantly, there are also indications that coinfection rates are increasing in India, typically seen amongst migrant labourers, who are traveling from the poor, rural VL-endemic areas to the major cities [9]. A recent study found 5.6% of VL cases aged ≥14 years to be coinfected [10]. However, there are currently no reliable estimates of VL-HIV burden in the most affected regions, given the lack of strong surveillance mechanisms.

VL-HIV coinfection is characterized by a number of complexities and clinical challenges. Serological tests of VL are less accurate in HIV coinfected individuals [3], [11]. Molecular tests are more accurate, but often not readily available in poor health care settings. Consequently, diagnosis often relies on invasive procedures, mainly by spleen or bone marrow aspiration. Treatment of VL-HIV coinfection is also extremely challenging, especially in eastern Africa. Case fatality rates are high, particularly in settings still relying on antimonials, reaching up to 25% [4]. In Ethiopia, 16% of primary VL and 56% of VL relapse cases demonstrated parasitological failure after treatment with liposomal amphotericin B at a total dose of 30 mg/kg [12]. Even after achieving initial parasitological clearance and initiation of antiretroviral treatment (ART), up to 60% of patients will relapse within a year [13].

There are a number of issues concerning interventions that are debated upon. For instance, whether or not secondary prophylaxis should be initiated in areas with anthroponotic transmission is still unclear [3]. Limited information is available on drug interactions between antiretroviral and antileishmanial drugs. The role of adjunctive immunotherapy against VL in HIV coinfection remains to be defined. Concerns have also been raised that coinfected individuals could be sources for the emergence and spread of drug-resistant *Leishmania* parasites [3], [14]. Despite its global emergence and important clinical and public health implications, the knowledge and operational gaps remain huge.

As the experience in Europe has shown, wide-scale introduction of ART can have a clear VL preventative effect. Achieving high ART coverage, early ART initiation (when CD4 counts are still high), and retention in care is thus an important goal for national programs. However, in many areas, VL-HIV coinfected individuals are highly mobile, with overall poor treatment access and high rates of loss to follow-up from HIV treatment programs. *L. donovani* in Ethiopia might also be more virulent than *L. infantum* in Europe [3]. Complementary preventative strategies targeting latent or the early stage of infection has successfully been implemented in other HIV-associated opportunistic infections such as tuberculosis and cryptococcosis. Such approaches merit exploration for VL-HIV coinfection as well [15].

From the purely scientific perspective, VL-HIV coinfection has a number of fascinating features. Its immunopathogenesis remains poorly understood. The commonly observed lack of immunological recovery despite VL treatment and HIV suppression with ART is unexplained. In contrast with tuberculosis, cryptococcal meningitis, and most other opportunistic infections, the immune reconstitution syndrome (IRIS) in VL-HIV seems exceptional [3]. A chronic/intermittent course of VL lasting several years has been described, labelled as “active chronic visceral leishmaniasis” [16]. Whether the poor immune recovery and associated VL relapse is primarily driven by parasite persistence or by an underlying immunological process is an open question [17].

How should we move on from here? What are the next steps? Research efforts and collaboration targeting VL-HIV coinfection should be intensified, especially in the hardest hit regions. Effectively tackling VL-HIV will require research organisations, clinicians, implementers, and other stakeholders to link up internationally. For instance, the AfriCoLeish Consortium, supported through the European Union, has recently been launched (http://www.africoleish.org). As part of its activities, two clinical trials (one on secondary prophylaxis, one on VL combination therapy) will be conducted in coinfected patients in Ethiopia.

The existence of an international network would create opportunities for improved global surveillance, exchange of expertise, and experience amongst stakeholders. Such a network can also foster standardization of research methodologies and bring along additional advantages in terms of advocacy and funding opportunities. Efforts can be pooled to improve access to VL care and treatment in resource-limited endemic countries, including the wider availability of drugs such as liposomal amphotericin B and miltefosine. Comprehensive research agendas and action plans could be drafted in a concerted effort. VL-HIV coinfection is a global problem in a globalized world; addressing the problem at the international level makes sense and requires setting up a global network.

Within such a network, reflections should be made on how to maximize the impact of current and future research efforts at the global level. Novel diagnostic tests need to be evaluated; biomarker studies, building on the 'omics technologies, will have to be conducted to develop novel diagnostic and prognostic markers or reveal new therapeutic avenues. To better understand the parasite and host factors, along with the immunological processes that characterize VL-HIV coinfection, cellular and molecular studies will be needed. To facilitate this, investment in systematic and standardized study designs and protocols, as well as sharing of bio specimens, might be a critical way to exponentially speed up the research process. The human African trypanosomiasis specimen bio bank could serve as inspiration [18]. However, ethical and regulatory aspects will require due consideration [19]–[21].

Importantly, research on VL-HIV coinfection should go beyond laboratory and clinical research, linking up with public health, environmental, and social sciences to engage in truly trans-disciplinary research initiatives. For instance, socioeconomic determinants driving VL-HIV coinfection and health-seeking behaviour of this specific population remain under-researched. Operational research will be required to optimize the implementation of (cost-)effective and sustainable disease control activities and move towards VL elimination.

In several African countries, VL care and treatment is still strongly driven by and dependent on non-governmental international agencies. Reinforcing national programs will be crucial. Similarly to tuberculosis and HIV coinfection [22], effective integration of national HIV and VL treatment programs should be pursued.

*PLOS Neglected Tropical Diseases* has dedicated a collection to the topic of VL-HIV coinfection. Such initiatives are highly valuable as they can help fill the knowledge gaps and can create a momentum for enhancing research and disease control efforts in this emerging public health problem. Progress towards elimination and control of VL and VL-HIV coinfection will ultimately hinge on the concerted efforts of all stakeholders, within a multidisciplinary approach, with research feeding into policy.
